# COX-2/sEH dual inhibitor PTUPB suppresses glioblastoma growth by targeting epidermal growth factor receptor and hyaluronan mediated motility receptor

**DOI:** 10.18632/oncotarget.20928

**Published:** 2017-09-15

**Authors:** Junyang Li, Yali Zhou, Handong Wang, Yongyue Gao, Liwen Li, Sung Hee Hwang, Xiangjun Ji, Bruce D Hammock

**Affiliations:** ^1^ Department of Neurosurgery, Jinling Hospital, Medical school of Nanjing University, Nanjing, 210002, China; ^2^ Department of Entomology and Nematology and UC Davis Comprehensive Cancer Center, University of California, Davis, CA 95616, USA

**Keywords:** cyclooxygenase-2, soluble epoxide hydrolase, epidermal growth factor receptor, hyaluronan mediated motility receptor, glioblastoma

## Abstract

**Aims:**

Cyclooxygenase-2 (COX-2)/soluble epoxide hydrolase (sEH) dual inhibitor, PTUPB, has been demonstrated to inhibit angiogenesis, primary tumor growth and metastasis. The aim of this study is to investigate the effects of PTUPB on glioblastoma cells and xenograft model.

**Results:**

We show here that PTUPB inhibits glioblastoma cell proliferation and G1 phase cell cycle arrest *in vitro*, and suppresses the tumor growth and angiogenesis *in vivo*. The expression and activation of epidermal growth factor receptor (EGFR) and its downstream kinases, ERK1/2 and AKT, are reduced by PTUPB, indicating that the EGF/EGFR signaling pathway is a potential target. Moreover, PTUPB dramatically suppresses expression of hyaluronan mediated motility receptor (HMMR) in the glioblastoma cell lines and xenograft mouse model, suggesting that the HMMR is the other potential target.

**Materials and Methods:**

Cellular immunofluorescence assays were used for cell staining of actin fibers and HMMR. CCK-8 kit was used for cell proliferation assay. Cell-cycle analysis was performed by flow cytometry. Quantitative real-time PCR assay was performed to test mRNA level. Western blot analysis was used to test protein expression. Immunohistochemical staining assay was used for xenograft tumor tissue staining of Ki-67, CD31 and HMMR. The SPSS version 17.0 software was applied for statistical analysis.

**Conclusions:**

Our data demonstrate that PTUPB is a potential therapeutic agent to treat glioblastomas.

## INTRODUCTION

Glioblastomas are the most malignant central nervous system (CNS) tumors in adults. Despite the recent advances in therapeutic strategies including surgery, radiotherapy and chemotherapy, the prognosis of glioblastoma patients remains poor with a median survival of less than one year [1–3]. As solid tumors, glioblastomas are highly vascularized and lead to rapid cell proliferation [4]. Thus, therapies of anti-angiogenesis and anti-tumorigenesis should be concurrently considered for glioblastoma treatment. Although an earlier study demonstrated that bevacizumab plus radiotherapy and temozolomide do not improve survival in glioblastoma patients [1], a recent exploratory analysis suggested that the addition of bevacizumab to standard glioblastoma treatment may extend survival for patients who do not receive second-line therapy [5].

Thanks to recent advances in tumor-microenvironment, increasing evidence reveals the close connections between inflammation and cancers [6–8]. The cyclooxygenase-2 (COX-2) signaling pathway in the arachidonic acid (ARA) cascade plays key roles in both inflammation and cancer [9]. Besides the anti-inflammatory and analgesic effects, COX-2 selective inhibitors (coxibs) also exert anti-tumorigenic effects on various cancers, including gliomas [10, 11]. Inhibition of COX-2 was shown to prevent tumor progression by blocking conversion of ARA to prostaglandin E2 (PGE2), a proinflammatory eicosanoid that promotes tumorigenesis and tumor-associated angiogenesis [12–14]. ARA can also be converted to epoxyeicosatrienoic acids (EETs) by cytochrome P450 (CYP) epoxygenases [15]. EETs are autocrine and paracrine lipid mediators that modulate ion transport and gene expression, and exert anti-inflammatory, analgesic, and cardioprotective effects. However, EETs are rapidly metabolized *in vivo* by soluble epoxide hydrolase (sEH) to dihydroxyeicosatrienoic acids (DHETs) which have no or less biological activity [16]. Therefore, sEH inhibitors (sEHIs) are used to stabilize endogenous EETs and thus preserve their biological activity [17]. Recently, Zhang, et al. [18] demonstrated that dual inhibition of COX-2 and sEH by a combination treatment synergistically suppresses lung tumor growth and metastasis. Then, COX-2/sEH dual inhibitors such as PTUPB were synthesized. Concurrent inhibition of both COX-2 and sEH using PTUPB as a single molecule dramatically suppresses angiogenesis and primary tumor growth and metastasis.

Given that the potential of PTUPB to suppress solid tumors by blocking angiogenesis, we investigated the effects of PTUPB on glioblastoma. In the present study, we demonstrated that PTUPB inhibits cell proliferation and G1 phase cell cycle arrest in glioblastoma cell lines, and suppresses both tumorigenesis and angiogenesis in glioblastoma xenografts. Moreover, we showed that PTUPB may exert anti-glioblastoma effects by suppressing expression of hyaluronan mediated motility receptor (HMMR) and by targeting epidermal growth factor receptor (EGFR) signaling pathway. Our data suggest that PTUPB may exert both anti-angiogenic and anti-tumorigenic effects on glioblastoma.

## RESULTS

### PTUPB suppresses glioblastoma growth *in vitro*

**Figure 1:**
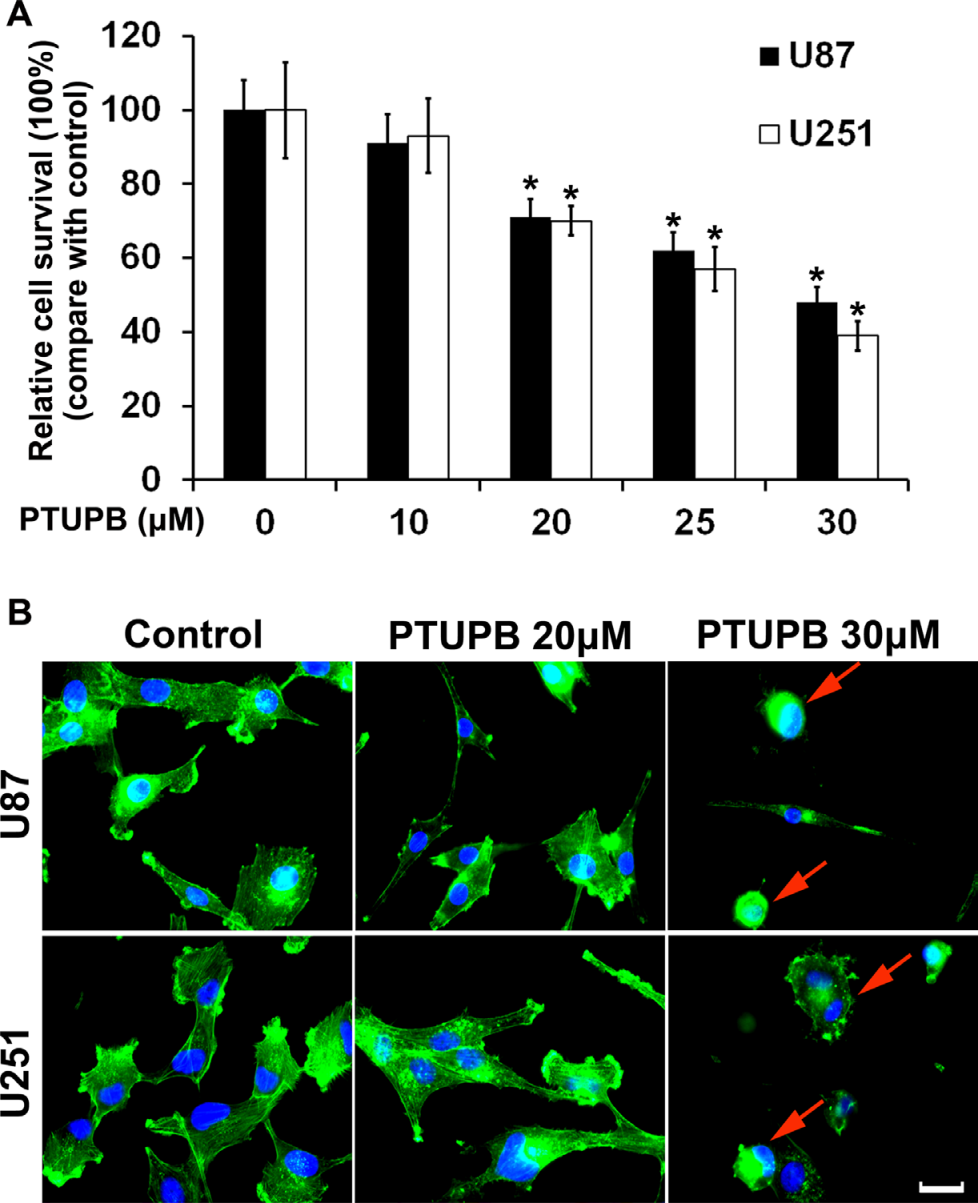
PTUPB inhibits cell proliferation and disrupts actin stress fibers (**A**) PTUPB inhibits human glioblastoma U87 and U251 cell proliferation in a concentration-dependent manner starting from 20 μM concentration (^*^P < 0.05, compare with the control group). (**B**) 30 μM PTUPB induces disruption of actin stress fibers and retraction of the cytoplasm in U251 and U87 cells. The actin stress fibers are shown in green and the nuclei are shown in blue. The red arrows point cell morphology change and cytoplasm retraction. Scale bar = 10 μm.

To investigate the effects of PTUPB on glioblastoma growth *in vitro*, U87 and U251 cells were treated with either DMSO (vehicle control) or PTUPB in various concentrations (10, 20, 25 or 30 μM) for 72 h. A cell counting kit-8 (CCK-8) was used to test the cell proliferation. PTUPB inhibited cell proliferation in a concentration-dependent manner (Figure 1A). By performing immunofluorescence staining with FITC-phalloidin, we found that 30 μM PTUPB causes disruption of actin stress fibers and retraction of the cytoplasm, which results in change in cell morphology and loss of adhesion to the substratum (Figure 1B). These observations indicate that PTUPB may exert a cytochalasin-like effect characterized by disruption of actin cytoskeleton, leading to growth inhibition and cytotoxicity.

**Figure 2:**
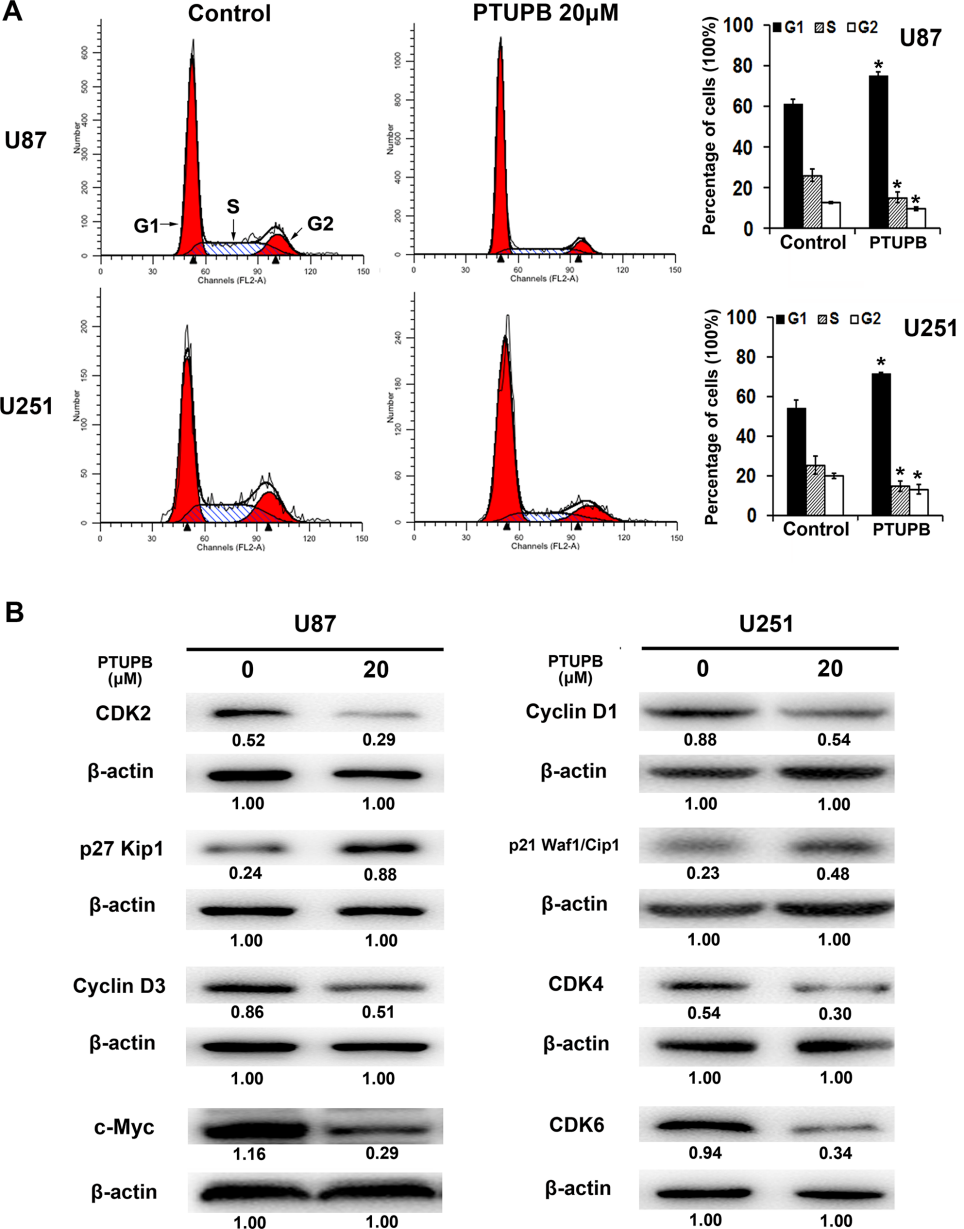
The effect of PTUPB on cell cycle (**A**) 20 μM PTUPB (48-h treatment) increases the percentage of G1 phase cells and decrease the percentage of S and G2 phase cells, compared with the control (^*^P < 0.05). (**B**) 20 μM PTUPB (48–h treatment) reduces the expression of cell cycle promoters CDK2, CDK4, CDK6, Cyclin D1, Cyclin D3 and c-Myc and, increases the expression of cell cycle progression inhibitors, p27^Kip1^ and p21^Waf1/Cip1^. β-actin served as loading control. The immunoblotting bands were quantified by ImageJ and represented by relative values compare with loading control (1.00).

Then, we performed cell cycle analysis and found that 20 μM PTUPB with 48 h treatment significant increases the percentage of G1 phase cells and conversely decreases the percentage of S and G2 phase cells (Figure 2A). Western blot also showed that PTUPB affects various cell cycle regulators for G1 to S phase transition. The expression of cell cycle promoters, CDK2, CDK4, CDK6, Cyclin D1, Cyclin D3 and c-Myc, were decreased, and the expression of cell cycle progression inhibitors, p27^Kip1^ and p21^Waf1/Cip1^, were increased (Figure 2B).

### PTUPB reduces both expression and phosphorylation of EGFR

**Figure 3:**
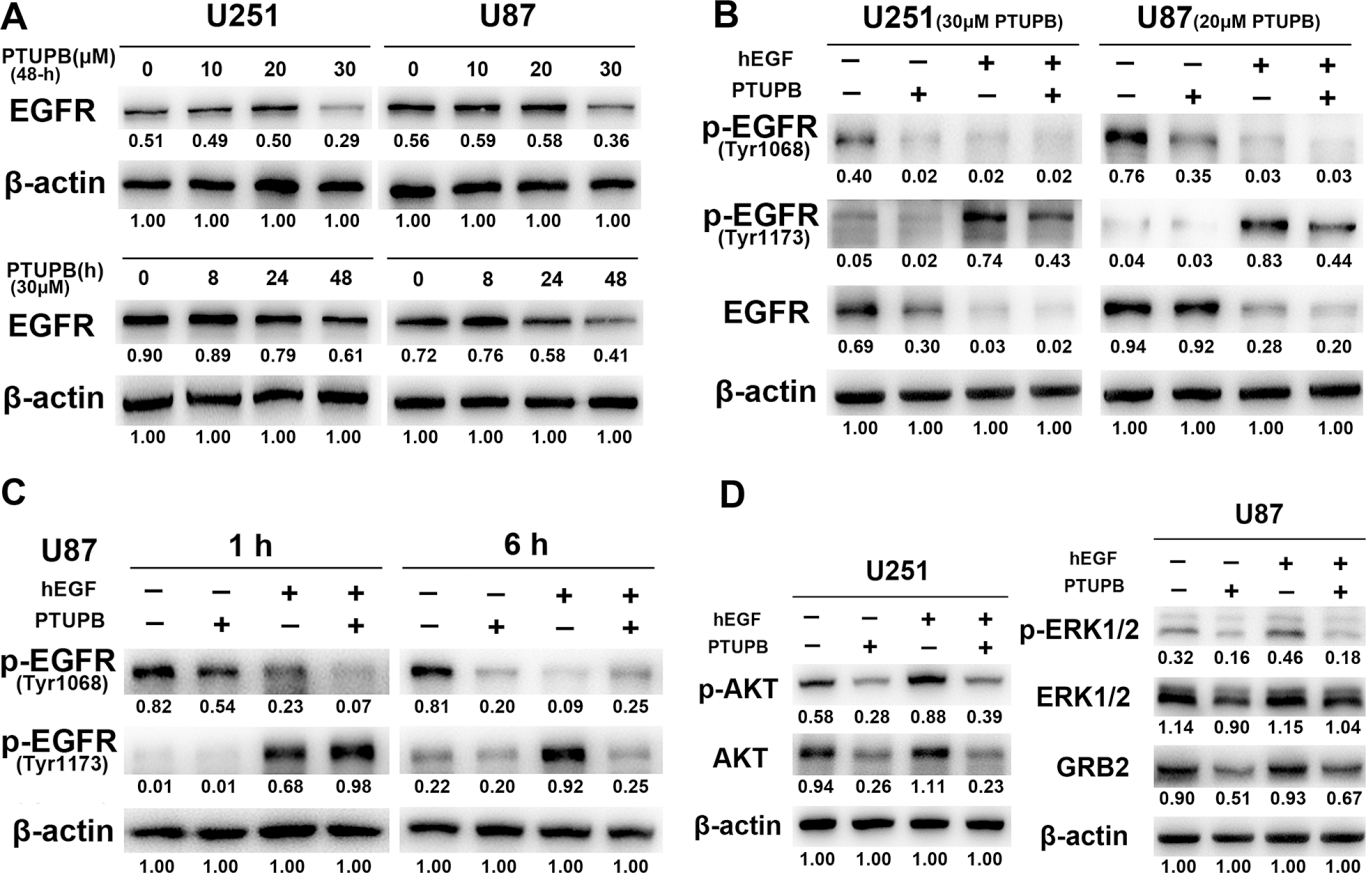
PTUPB targets EGFR signaling (**A**) When cells were treated for 48 h, a 30 μM PTUPB concentration was needed to significantly reduce EGFR expression. When cells were treated with 30 μM PTUPB, reduction in EGFR expression was seen until 24-h post treatment. (**B**) U251 cells were treated with 30 μM PTUPB or/and 50 ng/ml hEGF for 48 h and, U87 cells were treated with 20 μM PTUPB or/and 50 ng/ml hEGF for 48 h. 30 μM PTUPB reduces expression of EGFR and depletes p-EGFR^Tyr1068^ and p-EGFR^Tyr1173^. 20 μM PTUPB also depletes p-EGFR^Tyr1068^ and p-EGFR^Tyr1173^, but does not reduce EGFR protein expression. (**C**) In U87 cells, 1-h treatment of 30 μM PTUPB depletes p-EGFR^Tyr1068^ but not p-EGFR^Tyr1173^; while 6-h treatment of 30 μM PTUPB depletes p-EGFR^Tyr1173^ and p-EGFR^Tyr1068^ (50 ng/ml hEGF was used as an EGFR activator). (**D**) U251 and U87 cells were treated with 30 μM PTUPB in the presence or absence of 50 ng/ml hEGF for 48 h. PTUPB reduces the expression and phosphorylation of AKT and ERK1/2 and, the expression of GRB2. β-actin was used as a loading control. The immunoblotting bands were quantified by ImageJ and represented by relative values compare with loading control (1.00).

EGFR signaling plays key roles in cytoskeletal and cell cycle regulation, and cell growth promotion [19–21]. Thus, we investigated whether PTUPB exerts the inhibitory effects by targeting EGFR signaling pathway. Recombinant human epidermal growth factor (hEGF) was used as an EGFR activator. 30 μM PTUPB with either 24 h or 48 h treatment reduced the expression of EGFR protein (Figure 3A). Either 20 or 30 μM PTUPB with 48 h treatment could deplete phosphorylation of EGFR at both Tyr1068 (p-EGFR^Tyr1068^) and Tyr1173 (p-EGFR^Tyr1173^) (Figure 3B). We also found that PTUPB depletes p-EGFR^Tyr1068^ in the early phase (1 h treatment) but p-EGFR^Tyr1173^ in the late phase (6 h) (Figure 3C). The expression of GRB2, an adaptor protein that directly binds to p-EGFR^Tyr1068^ and regulates EGFR/MAPK signaling, was also reduced, and the expression and phosphorylation of ERK1/2 and AKT were suppressed by PTUPB (Figure 3D).

### PTUPB suppresses the expression of HMMR and stemness markers and regulators

**Figure 4:**
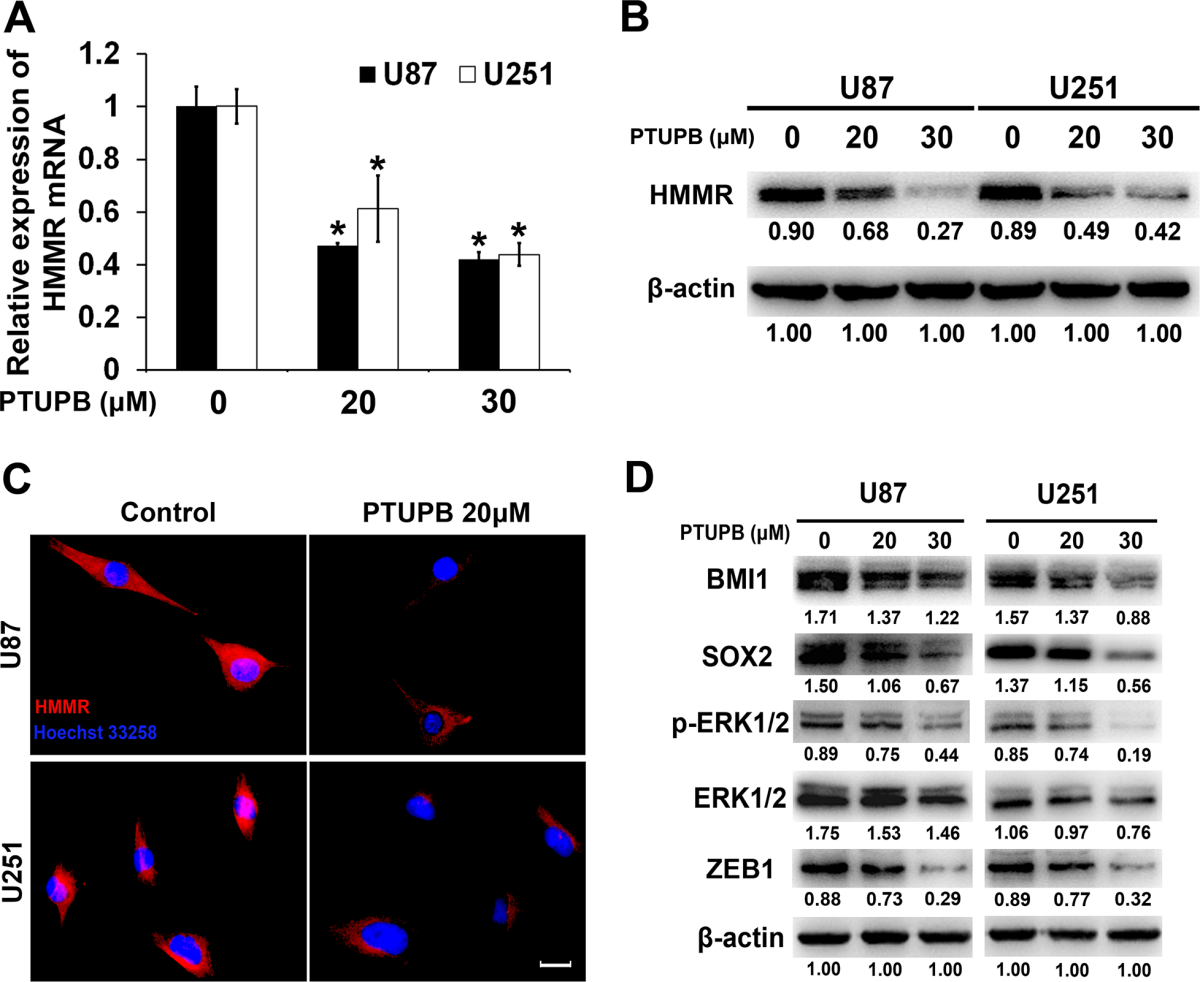
PTUPB suppresses expression of HMMR and stemness markers and regulators (**A**) Quantitative real time-PCR assay showed that HMMR mRNA level is reduced by PTUPB (^*^P < 0.05). The western blot assay (**B**) or cellular immunofluorescence (**C**) Scale bar = 10 μm) showed that HMMR protein level is significantly reduced by PTUPB. (**D**) The western blot assay showed that PTUPB suppresses the expression of BMI1, SOX2 and ZEB1 and the activation of ERK1/2. β-actin was used as a loading control. The immunoblotting bands were quantified by ImageJ and represented by relative values compare with loading control (1.00).

After treating both U251 and U87 cells with either DMSO (vehicle control) or PTUPB, the expression of HMMR mRNA level was determined by quantitative real time-PCR assay (Figure 4A) and HMMR protein was tested by western blot and cellular immunofluorescence (Figures 4B and 4C). Both protein and mRNA level of HMMR is remarkably suppressed by PTUPB in a concentration dependent manner.

Targeting HMMR has been demonstrated to inhibit the expression of stemness markers and regulators and the activation of ERK1/2 [22]. We tested the expression of BMI1, SOX2 and ERK1/2 in PTUPB treated U251 and U87 cells, and found that PTUPB significantly inhibits the expression of BMI1, SOX2 and the activation of ERK1/2 (Figures 4D). ZEB1 is a target of SOX2 and has been shown to be co-expressed with SOX2 in glioblastoma [23]. We then tested the expression of ZEB1 in PTUPB treated U251 and U87 cells, and found that PTUPB also inhibits expression of ZEB1 (Figures 4D).

### PTUPB suppresses glioblastoma growth *in vivo*

**Figure 5:**
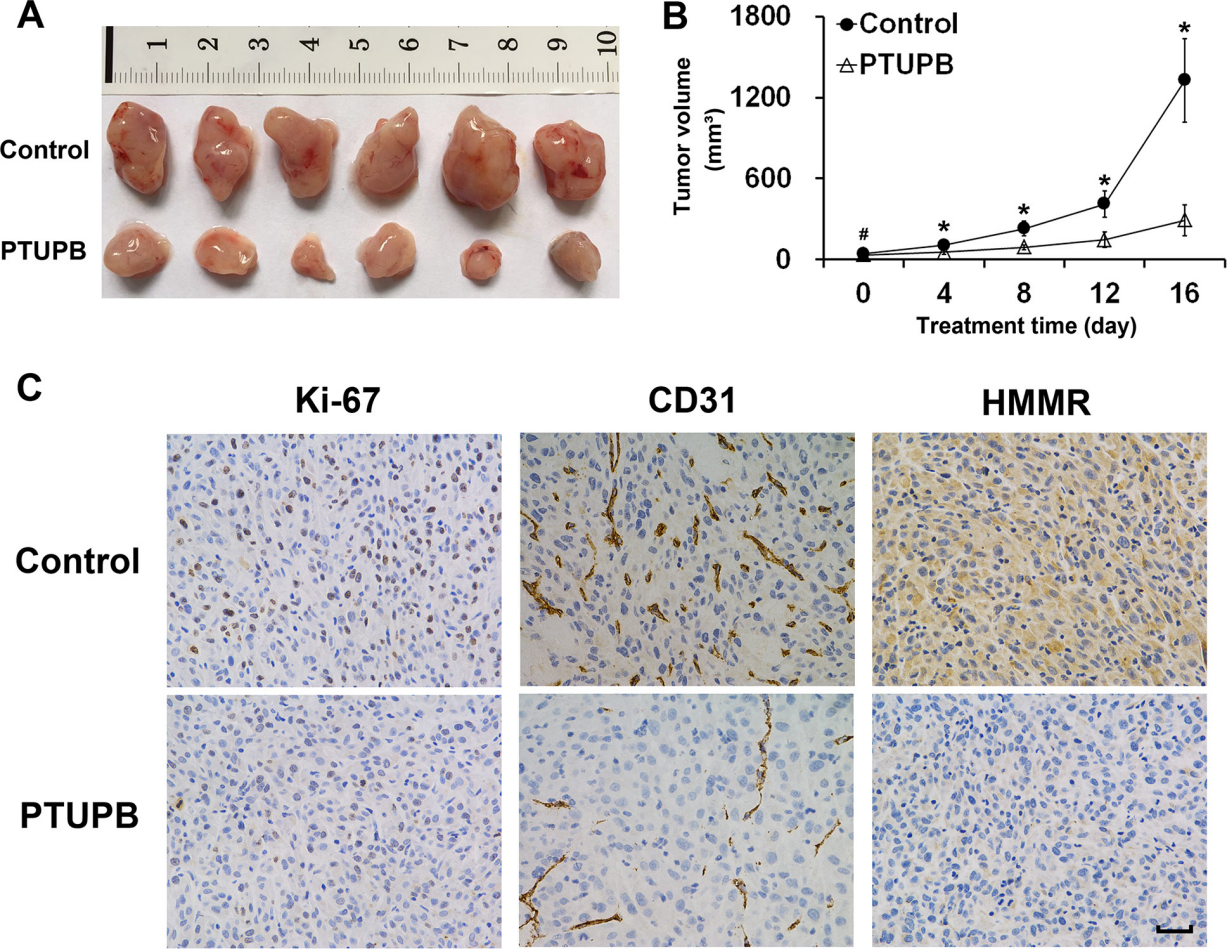
PTUPB suppresses glioblastoma growth *in vivo* (**A**) Xenograft tumors were obtained from mouse models (*n* = 6 mice per group). (**B**) Tumor bearing mice were treated with either vehicle control (1:1 (v/v) PEG 400/DMSO) or PTUPB (60 mg·kg^−1^ d^−1^, dissolved in 1:1 (v/v) PEG 400/DMSO), and the tumor volumes were measured (^#^*P* > 0.05, ^*^P < 0.05). (**C**) The immunohistochemical staining shows that PTUPB suppresses expression of Ki-67, CD31 and HMMR in xenograft tumors. Ki-67 is positively stained in nuclei and, HMMR is generally stained in cytoplasm. CD31 is positively stained in vascular endothelial cell. Scale bar = 50 μm.

To investigate the effects of PTUPB on glioblastoma *in vivo*, we built a subcutaneous glioblastoma xenograft model in BALB/c nude mice. As shown in Figure 5A and 5B, PTUPB significantly suppressed tumor growth. Then, we dissected the xenograft tumors for immunohistochemical staining. As seen in Figure 5C, PTUPB lowered expression of Ki-67, CD31 and HMMR. Ki-67 is a key biomarker of cancer cell proliferation and HMMR plays key roles in maintaining tumor growth and malignance. Lower CD31 expression in PTUPB-treated tumors represents the inhibition of tumor angiogenesis. Thus, our data indicated that PTUPB inhibits glioblastoma growth *in vivo*.

## DISCUSSION

COX-2 is activated in many cancers and contributes to poor prognosis of malignances by enhancing resistance to chemotherapy and/or radiotherapy [24–26]. Therefore, targeting COX-2 has been demonstrated to be effective in treating cancers, including malignant gliomas [10]. Meanwhile sEH inhibitors have been demonstrated to reduce cardiovascular side effects of coxibs [27] and gastrointestinal erosion associated with COX inhibition [28]. sEHIs also potentiate anti-inflammatory and antiangiogenic effects of coxibs [18, 29–31]. Dual inhibition of COX-2 and sEH by either a combination of selective inhibitors of each enzyme or a COX-2/sEH dual inhibitor PTUPB as a single agent suppress primary tumor growth and metastasis by inhibiting tumor angiogenesis with the mechanisms of reduction of VEGF and PGE2, stabilization of EETs and inhibition of endothelial cell proliferation [18].

In the present study, the effects of PTUPB on human glioblastoma cells and the xenograft model were tested. We found that 20 μM PTUPB significantly inhibits cell proliferation and induces G1 phase cell cycle arrest in multiple glioblastoma cell lines. Then, we performed immunofluorescence staining of actin stress fibers with FITC-phalloidin to present the morphology of cells treated. 30 μM PTUPB significantly disrupts cellular actin cytoskeleton and induces retraction of the cytoplasm and loss of cell adhesion to the substratum, suggesting that PTUPB possesses cytochalasin-like anti-actin effect that leads to cell growth inhibition and following cell death [32]. Although 20 μM PTUPB inhibits cell proliferation, it does not disrupt actin cytoskeleton, indicating that PTUPB may induce more dramatic cell growth inhibition by disrupting the actin and inducing loss of cell adhesion.

Next, we studied whether PTUPB affects EGFR, a critical receptor tyrosine kinase that promotes glioblastoma angiogenesis and increases glioblastoma cell proliferation, migration and invasion [33–37]. EGFR is overexpressed in 50–60% of glioblastomas and is amplified in 40% of the tumors, and half of these contain various EGFR mutations [34, 36]. Several tyrosine kinase inhibitors (TKIs) that target the EGFR have been evaluated in clinical trials for glioblastoma patients, but all have failed to meet expectations [34, 38]. In the present study, we demonstrated that PTUPB depletes activated EGFR (p-EGFR^Tyr1068^ and p-EGFR^Tyr1173^) and reduces protein levels of EGFR and the expression and activation of its downstream kinases ERK1/2 and AKT. In the U251 and U87 glioblastoma cell lines, p-EGFR^Tyr1068^ is spontaneously expressed, while EGF-induced p-EGFR^Tyr1173^ is persistent (no less than 48 h). The high levels of p-EGFR^Tyr1068^ and p-EGFR^Tyr1173^ represent the hyper-activity of EGFR signaling, but it is dramatically inhibited by PTUPB. Binding of GRB2 to the p-EGFR^Tyr1068^ is crucial to the EGFR-induced MAPK signaling pathway [39]. Our data show that PTUPB also depletes GRB2 expression. We also found that 20 μM PTUPB dramatically depletes p-EGFR, although it does not reduce the EGFR protein level. This result may partially support the G1 phase arrest induced by 20 μM PTUPB. Unlike the ATP-competitive TKIs, PTUPB treatment does not deplete EGF-induced phosphorylation of EGFR (p-EGFR^Tyr1173^) until 6 h post dose, while Gefitinib, a widely-used EGFR TKI, inhibits EGF-induced p-EGFR in early phase (30 min) in glioblastoma cells [37]. Thus, we hypothesize that PTUPB may exert anti-EGFR effects by depleting phosphorylated-EGFR rather than blocking EGFR phosphorylation in an ATP-competitive mechanism. This different mechanism of action by PTUPB targeting EGFR may offer a supplementary strategy to avoid TKI-resistance. Nonetheless, how PTUPB affects the protein structure of EGFR remain unclear, and further studies are needed to unveil the mechanisms.

Then, we found that in both glioblastoma cells and xenografts PTUPB suppresses expression of HMMR, a multifunctional oncogenic protein which is highly expressed in malignant tumors [40–44]. HMMR not only regulates tumor cell proliferation and invasion, but also affects centrosome structure and regulates mitotic spindle formation [45, 46]. HMMR maintains the stemness and tumorigenicity of stem-like cells, which is essential for promoting tumorigenesis and angiogenesis in glioblastoma. Targeting HMMR has been demonstrated to inhibit the expression of stemness markers and regulators and the activation of ERK1/2 [22]. In the present study, we demonstrated that PTUPB also inhibits the expression of stemness maerkers BMI1, SOX2 and the activation of ERK1/2 in glioblastoma cell lines. Moreover, we found that the ZEB1 protein, a member of the ZEB family of zinc finger transcription factors, was dramatically suppressed by PTUPB. Very recently, ZEB1 has been identified as a potential SOX2 target, which is co-expressed with SOX2 in glioblastoma [23]. Our findings indicated that PTUPB may target the HMMR/SOX2/ZEB1 signaling axis, inhibiting glioblastoma growth.

In conclusions, we found that the COX-2/sEH dual inhibitor PTUPB suppresses human glioblastoma growth *in vitro* and *in vivo*, and dramatically inhibits EGFR signaling pathway and expression of HMMR and stemness markers and regulators. Therefore, PTUPB may be a potential therapeutic agent to treat glioblastomas.

## MATERIALS AND METHODS

### Reagents

The cyclooxygenase (COX)-2/soluble epoxide hydrolase (sEH) dual inhibitor, PTUPB, was synthesized by Dr. Sung Hee Hwang as described [18, 47]. The recombinant human EGF (hEGF) (#8916) and primary antibodies against β-actin (#3700), CDK2 (#2546), p27^Kip1^ (#3686), Cyclin D3 (#2936), c-Myc (#13987), Cyclin D1 (#2978), p21^Waf1/Cip1^ (#2947), CDK4 (#12790), CDK6 (#3136), EGFR (#4267), p-EGFR^Tyr1068^ (#3777), p-EGFR^Tyr1173^ (#4407), AKT (#2920), p-AKT^Ser473^ (#4060), ERK1/2 (#4695), p-ERK1/2^Thr202/Tyr204^ (#4377), GRB2 (#3972), BMI1 (#6964), ZEB1 (#3396) and SOX2 (#3579) were all purchased from Cell Signaling Technology (Beverly, MA, USA). Antibody against HMMR (#GTX121502) was purchased from GeneTex (Irvine, CA, USA).

### Cell culture

Human glioblastoma cell lines U251 and U87 were purchased from CBTCCCAS (Cell Bank, Type Culture Collection of Chinese Academy of Sciences). All cells were cultured at 37°C in a humidified atmosphere of 95% air and 5% CO_2_ by using the complete culture medium, Dulbecco's Modified Eagle's Medium (DMEM) supplemented with 10% fetal bovine serum (FBS) and 1% penicillin and streptomycin.

### Cellular immunofluorescence assays

Cells were transplanted into 6-well plates covered with circular coverslips in the bottom and allowed to attach overnight. After different treatment of PTUPB for 48 h, cells were fixed by 4% paraformaldehyde. For phalloidin staining of actin fibers, fixed cells were incubated with fluorescein isothiocyanate (FITC)-labeled phalloidin (Beyotime Institute of Biotechnology, Shanghai, China) for 60 min at room temperature and then mounted onto slides for observation after nuclei staining. For HMMR staining, fixed cells were probed with HMMR primary antibody (OriGene, Rockville, MD, USA) at 4°C overnight followed by incubation with Alexa Fluor 555-labeled secondary antibody (Beyotime Institute of Biotechnology, Shanghai, China) for 60 min at room temperature. Hoechst 33258 was used for staining nuclei. Cell samples were observed and pictures were taken by using a Zeiss Axio Scope.A1 microscope (Carl Zeiss, Oberkochen, Germany).

### Cell proliferation assay

CCK-8 kit purchased from Dojindo Laboratories (Kumamoto, Japan) was used for cell proliferation assay. Cells were transplanted into 96-well plates followed by different treatment. After 72 h, culture medium was discarded; cells were then cultured in 100 μl fresh serum-free medium contained 10 μl CCK-8 solutions for 2 h. Then, the 96-well plate was put into an enzyme-linked immunosorbent assay plate reader (Bio-Rad Laboratories, Inc., Berkeley, CA, USA) and optical density values (absorbance) were recorded at 450 nm. The relative cell survival rate was calculated according to the absorbance.

### Cell cycle analysis by flow cytometry

As we described previously [48], the cell cycle analysis was performed using a FACS Calibur flow cytometer (BD Biosciences, San Jose, CA, USA). Briefly, cells were treated with either 20 μM PTUPB or DMSO and harvested by trypsinization, centrifuged (3500 rpm for 5 min), and washed twice with PBS. Cells were then fixed by 75% ethanol. After staining using propidium iodide (PI), a total of 10,000 nuclei were analyzed.

### Quantitative real-time PCR assay

Total RNA was extracted from cells samples by Trizol (Invitrogen) according to the manufacturer's instruction. Then, the RNA was reverse transcribed to form cDNA by SuperScript III Reverse Transcriptase (Invitrogen). ABI 7300 real-time PCR system (Applied Biosystems) was applied to perform real-time PCR using Fast Start Universal SYBR Green Master (Roche). The primers were: HMMR (Forward: CATGGTGCAGC TCAGGAACA; Reverse: AAGCTGACAGCGGAGTTT TG); β-actin (Forward: CACCCAGCACAATGAAGATCA AGAT; Reverse: CCAGTTTTTAAATCCTGAGTCAA GC). For data analysis, the ΔΔCt (comparative threshold cycle) method was used. The fold-change was calculated as 2^−ΔΔCt^. The average expression of housekeeping genes (β-actin) was used to normalize the data.

### Western blot analysis

As we described previously [48], the whole cell lysates were prepared and separated by SDS-polyacrylamide gel electrophoresis (SDS-PAGE) and transferred to a polyvinylidene fluoride membrane (Millipore Corporation, Bedford, MA, USA). Membranes were incubated with primary antibodies at 4°C overnight followed by incubation with secondary antibody. Immunoblots of proteins were visualized with chemiluminescence luminol reagents (Beyotime Institute of Biotechnology, Shanghai, China). Public software ImageJ (National Institutes of Health, USA) was used to quantify the densitometry of the immunoblotting bands.

### Tumor xenograft mouse model

Animal experiments protocols were approved by Jinling Hospital animal studies committee. Approximately 5.0 × 10^6^ U87 cells were transplanted subcutaneously to BALB/c nude mouse (male at 5 to 6 weeks old) to develop a mouse xenograft model of human glioblastoma. Once tumor was palpable, the treatment of PTUPB (60 mg·kg^−1^·d^−1^, dissolved in a mixed solvent of PEG-400 and DMSO, 1:1 vol/vol) or vehicle control (PEG-400 and DMSO, 1:1 vol/vol) was administered once daily by intraperitoneal injection. The length and width of the tumors were measured every other day using a vernier caliper, and the tumor volume was calculated (tumor volume = 1/2×length×width^2^). 16 days after the treatment, all tumors obtained from animals were submitted for immunohistochemical staining to analyze the expression of Ki-67, CD31 and HMMR.

### Immunohistochemical staining

Xenograft tumor tissues were obtained, paraffin-embedded and tissue sections prepared. Briefly, specimen was cut as 4 μm-thick sections, which were then deparaffinized. 3% H_2_O_2_ was used to quench endogenous peroxidase activity. Nonspecific bindings were blocked by normal goat serum. Immunostaining was performed using primary antibodies at either 1:100 or 1:200 dilution followed by incubation with HRP-labeled secondary antibody. The visualization signal was developed with 3,3′-diaminobenzidine (DAB), and then mounted. Tissue sections were observed and pictures were taken by using a Zeiss Axio Scope.A1 microscope (Carl Zeiss, Oberkochen, Germany).

### Statistical analysis

The SPSS 17.0 software and Microsoft Excel were used for statistical analysis. Comparisons between treated and control groups were carried out using one-way ANOVA or independent *t-test*, and expressed as mean ± standard deviation (SD). *P* < 0.05 were considered statistically significant.

## Abbreviations

CNS: central nervous system
COX-2: cyclooxygenase-2
ARA: arachidonic acid
PGE2: prostaglandin E2
EETs: epoxyeicosatrienoic acids
sEHIs: soluble epoxide hydrolase inhibitors
DHETs: dihydroxyeicosatrienoic acids
HMMR: hyaluronan mediated motility receptor
EGFR: epidermal growth factor receptor
DMSO: dimethyl sulfoxide
DMEM: Dulbecco's Modified Eagle's Medium
FBS: fetal bovine serum
CCK-8: cell counting kit-8
FITC: fluorescein isothiocyanate
PI: propidium iodide.
